# Characterization of the transcriptome and temperature-induced differential gene expression in QPX, the thraustochytrid parasite of hard clams

**DOI:** 10.1186/1471-2164-15-245

**Published:** 2014-03-28

**Authors:** Ewelina Rubin, Arnaud Tanguy, Mickael Perrigault, Emmanuelle Pales Espinosa, Bassem Allam

**Affiliations:** 1 School of Marine and Atmospheric Sciences, Stony Brook University, Stony Brook, NY 11794-5000, USA; 2 UPMC Université Paris 6, UMR 7144, Equipe Génétique et Adaptation en Milieu Extrême, Station Biologique de Roscoff, 29682 Roscoff, France

**Keywords:** Quahog Parasite Unknown (QPX), Virulence factors, Oligoarrays, Gene expression

## Abstract

**Background:**

The hard clam or northern quahog, *Mercenaria mercenaria*, is one of the most valuable seafood products in the United States representing the first marine resource in some Northeastern states. Severe episodes of hard clam mortality have been consistently associated with infections caused by a thraustochytrid parasite called Quahog Parasite Unknown (QPX). QPX is considered as a cold/temperate water organism since the disease occurs only in the coastal waters of the northwestern Atlantic Ocean from Maritime Canada to Virginia. High disease development at cold temperatures was also confirmed in laboratory studies and is thought to be caused predominantly by immunosuppression of the clam host even though the effect of temperature on QPX virulence has not been fully investigated. In this study, the QPX transcriptome was sequenced using Roche 454 technology to better characterize this microbe and initiate research on the molecular basis of QPX virulence towards hard clams.

**Results:**

Close to 18,000 transcriptomic sequences were generated and functionally annotated. Results revealed a wide array of QPX putative virulence factors including a variety of peptidases, antioxidant enzymes, and proteins involved in extracellular mucus production and other secretory proteins potentially involved in interactions with the clam host. Furthermore, a 15 K oligonucleotide array was constructed and used to investigate the effect of temperature on QPX fitness and virulence factors. Results identified a set of QPX molecular chaperones that could explain its adaptation to cold temperatures. Finally, several virulence-related factors were up-regulated at low temperature providing molecular targets for further investigations of increased QPX pathogenicity in cold water conditions.

**Conclusions:**

This is one of the first studies to characterize the transcriptome of a parasitic labyrinthulid, offering new insights into the molecular bases of the pathogenicity of members of this group. Results from the oligoarray study demonstrated the ability of QPX to cope with a wide range of environmental temperatures, including those considered to be suboptimal for clam immunity (low temperature) providing a mechanistic scenario for disease distribution in the field and for high disease prevalence and intensity at low temperature. These results will serve as basis for studies aimed at a better characterization of specific putative virulence factors.

## Background

QPX (Quahog Parasite Unknown) is a unicellular eukaryote that infects hard clam (*Mercenaria mercenaria*) populations along the northeastern coast of North America [1-4]. QPX is thought to be an opportunistic pathogen occurring at low abundance in coastal waters, sediments and organic debris [5-7], but occasionally becoming invasive to clams exhibiting lower resistance [8-10], suppressed immunity and/or during unfavorable environmental conditions [11-13]. QPX is an undescribed species belonging to thraustochytrids in the phylogenetic group *Labyrinthulomycetes*[14-16]. Labyrinthulids (slime nets) are composed of ecologically important but greatly understudied marine protists closely related to water molds (oomycetes), diatoms, golden and brown algae, all of which collectively form a monophyletic cluster named stramenopiles [17].

Labyrinthulids are marine saprophytes associated with marine algal and animal detritus, but some are found as parasitic, commensal and mutualistic species [18]. *Labyrinthula zostera* is the most known labyrinthulid parasite which has caused extensive devastation of marine seagrasses worldwide [19]. In the past, several labyrinthulids were reported to cause disease in mollusks including abalones, squids and nudibranchs [20-22]. In recent years, new labyrinthulid species have been emerging as parasites in other marine invertebrates [23-26]. All of these parasitic labyrinthulids lack any significant genomic or transcriptomic sequence information, making it difficult to determine how they evolved their parasitic abilities, and what the virulence mechanisms are which enable them to invade and survive in host tissues.

Even though significant clam mortality has been associated with QPX disease since the early 1950’s [3], many aspects of the disease remain unexplored and understudied. Little progress has been made to understand QPX basic cell biology, including its life stages, nutritional requirements, and factors mediating its virulence toward clams. As is often the case for opportunistic infections, QPX disease occurrence and development is significantly dependent upon external environmental conditions affecting both the parasite and the host. QPX’s natural geographic range of distribution shows that this parasite can be considered as a temperate/cold species, as it has not been described in the relatively warm waters south of Virginia. Interestingly, QPX growth *in vitro* is optimal between 20°C and 23°C, and growth decreases at both colder (below 8°C) and warmer (above 27°C) temperatures [27]. The major impact of temperature on disease development is reflected in the seasonal fluctuations of disease prevalence in clams, with peaks ranging from fall in MA to early summer and spring in NY and VA, respectively [1-4]. On the other hand, experimental transmission studies showed maximal disease development at low water temperatures, which has been attributed to the suppression of clam immunity [11,12] and it is hypothesized that immunosuppression is a major factor controlling QPX disease development. In fact, the effect of temperature on clam immunity was demonstrated [12] providing a possible mechanism for increased disease development at cold temperature. On the contrary, very little is known about the effect of temperature on QPX physiology, fitness or its virulence characteristics and ability to cause disease. Very recently, the partial sequencing of the QPX genome and two transcriptomes of the parasite provided some sequence resources for QPX investigations [28]. The present study was also conducted to gather transcriptomic sequence information for a better understanding of QPX biology and exploration of its virulence factors. For that purpose, transcriptome sequences of QPX were generated, assembled and annotated and then used to build a custom designed 15 K oligonucleotide array that was subsequently used to investigate QPX molecular response to temperature conditions known to regulate disease development [11,12]. Findings were discussed with a focus on the characterization of possible links between temperature and the expression profiles of virulence-related factors.

## Results and discussion

### Characterization of QPX transcriptome

In the present study, we sequenced, assembled and annotated the transcriptome of quahog parasite unknown, the thraustochytrid protist that causes disease and mortality in an important bivalve species, the hard clam *Mercenaria mercenaria*. The cDNA sequence library was generated from QPX cells (isolated from Raritan Bay, New York) grown in a variety of culture media to obtain satisfactory coverage for transcripts that may have different expression profiles under various culture conditions. A total of 490,726 sequence reads (average length of 215 bp) were generated by the 454 GS-FLX Titanium platform. Quality and length filtering yielded 223,652 high-quality reads that were assembled using MIRA. The resulting QPX transcriptome library consists of 17,934 transcript sequences including contigs (continuous sequences, 14,636), singletons (single copy sequences, 1,636), and long repetitive contigs (lrc, 1,662) with sequence length ranging from 40 bp to over 2,700 bp and a total of 7,111,495 bp in assembled library. Sequences longer than 200 bp have been deposited into DDBJ/EMBL/GenBank under the TSA (Transcript Shotgun Assembly) accession number: GALJ00000000, BioProject accession number: PRJNA207819, BioSample accession number: SAMN02194536, and Sequence Read Archive accession number: SRR900258. The number of assembled bases produced in this study represent about 20% of the genomic sequence assembly (34,620,595 bp assembled into 21,280 contigs) recently produced for QPX [28] which represents a smaller transcriptome coverage than that of other stramenopiles with fully sequenced genomes such as *Thalassiosira pseudonana* for which about 32% of the genome is represented by coding sequences [29].

**Figure 1:**
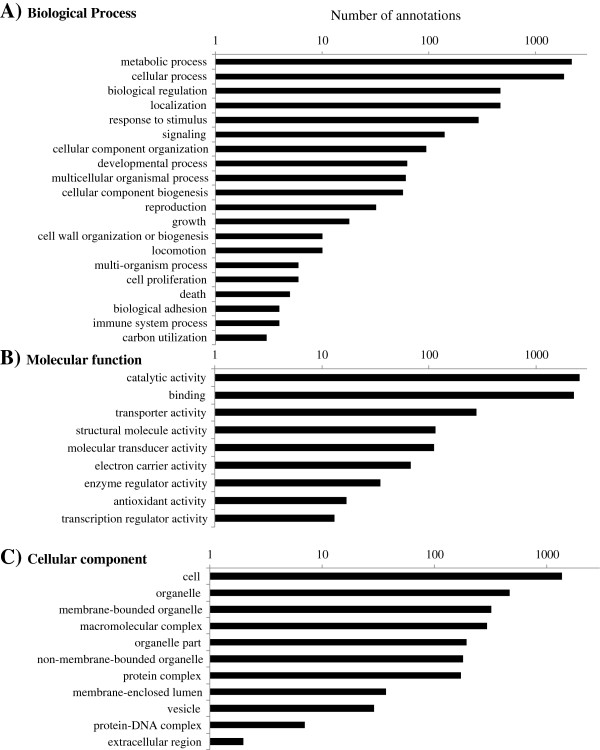
**Gene Ontology (GO) annotations of the QPX transcriptome.** GO phrases identified by the Blast2GO search tool in QPX transcriptome sequence library **A)** a total of 5,768 level-2 annotations within the biological process ontology; **B)** 5,459 molecular function level-2 annotations **C)** combined level-2 and level-3 annotations (3,081) within the cellular component ontology.

Blast2GO server was used to find homologous sequences to QPX transcripts in publicly available databases and to assign functional information to these transcripts. A total of 7,093 transcripts were found to have positive blastx homology matches (E < 10^-3^) to other sequences in NCBI database (Additional file 1). The top four species showing the most similarity to QPX sequences were the oomycete *Phytophthora infestans*, the brown alga *Ectocarpus siliculosus,* and the diatoms *Phaeodactylum tricornutum* and *Thalassiosira pseudonana*, all of which are stramenopilan species, thus confirming QPX affiliation to this phylogenetic group [17]. A total of 4,644 sequences were identified to have a match to at least one conserved protein domain in the InterPro database (Additional file 1) and 5,764 sequences were assigned at least one functional annotation term based on the Gene Ontology vocabulary database with a cut-off E-value of 10^-3^ (Additional file 1). Summary of the GO annotation is present in Figure 1. The level-2 functional annotation of QPX transcriptome returned 5,768 and 5,459 gene ontology phrases associated with the biological process (Figure 1A) and molecular function (Figure 1B) categories, respectively. The combined level 2 and 3 functional annotation of QPX transcripts resulted in 3,081 cellular component gene ontologies (Figure 1C).

### Putative virulence factors in QPX transcriptome

The primary goal of generating a transcriptome library for QPX was to provide resources for future studies on QPX virulence factors. The GO terms are commonly used as clues in the discovery of molecules involved in the infection process, pathogenicity and host-pathogen interactions [30]. Likely the most destructive virulence molecules are hydrolytic enzymes produced by pathogenic organisms to destroy host-derived biopolymers (proteins, polysaccharides and lipids) and to invade and colonize their hosts [31]. The annotation carried out in this study revealed 777 QPX transcripts encoding hydrolytic enzymes including glycosyl hydrolases (pectinase, amylases, cellulases, xylanases, chitnases, glucanases), peptidases, lipases and phospholipases (Table 1, Additional file 1), all of which have been found to be involved in pathogenicity of many microorganisms [32,33]. For example, chitinases from the oomycete pathogen *Aphanomyces astaci* of freshwater crayfish have been investigated as potential virulence factors [34]. Since the molluscan shell is partially composed of chitin [35,36] and QPX cells have been previously found associated with empty molluskan shells [5], QPX’s ability to digest shell material represents a venue for future investigations. Similarly, phospholipases are important enzymes utilized by many pathogenic organisms, including bacteria, fungi and protozoans, to hydrolyze cell membranes of host cells [37-41].

**Table 1:** Examples of gene ontology terms commonly associated with virulence identified in QPX transcriptome; F: molecular function, P: biological process

**Gene Ontology term**	**GO category**	**GO level**	**GO ID**	**Seqs no.**
**Degradation of biopolymers hydrolase activity**				
**Hydrolase activity**	**F**	**3**	**GO:0016787**	**777**
Nuclease activity	F	5	GO:0004518	29
Phosphatase activity	F	6	GO:0016791	27
Lipase activity	F	5	GO:0016298	9
Phospholipase activity	F	6	GO:0004620	4
Chitinase activity	F	6	GO:0004568	3
Glucosidase activity	F	6	GO:0015926	2
Amylase activity	F	6	GO:0016160	1
Galactosidase activity	F	6	GO:0015925	1
**Peptidase activity**	**F**	**4**	**GO:0008233**	**203**
Metallopeptidase activity	F	6	GO:0008237	47
Serine-type peptidase activity	F	5	GO:0008236	45
Cysteine-type peptidase activity	F	6	GO:0008234	35
Metalloendopeptidase activity	F	7	GO:0004222	30
Aspartic-type endopeptidase activity	F	7	GO:0004190	10
Threonine-type peptidase activity	F	6	GO:0070003	7
Peptidase inhibitor activity	F	4	GO:0030414	9
**Cell homeostasis and protection against oxidative stress**				
**Antioxidant activity**	**F**	**2**	**GO:0016209**	**17**
Peroxidase activity	F	3	GO:0004601	10
Superoxide metabolic process	P	5	GO:0006801	5
Catalase activity	F	4	GO:0004096	4
Thioredoxin-disulfide reductase activity	F	3	GO:0004791	2
Peroxiredoxin activity	F	5	GO:0051920	2
Thioredoxin peroxidase activity	F	4	GO:0008379	1
Glutathione peroxidase activity	F	4	GO:0004602	1
**Secretion and polysaccharide production**				
Polysaccharide metabolic process	P	4	GO:0005976	21
Polysaccharide biosynthetic process	P	6	GO:0000271	13
Lipopolysaccharide biosynthetic process	P	6	GO:0009103	3
Extracellular polysaccharide metabolic process	P	6	GO:0046379	2
Secretion	P	5	GO:0046903	8
Exocytosis	P	6	GO:0006887	4
Vesicle docking involved in exocytosis	P	5	GO:0006904	3
Vesicle-mediated transport	P	5	GO:0016192	38
**Adhesion and recognition**				
Cell adhesion	P	3	GO:0007155	4
Receptor activity	F	4	GO:0004872	34
G-protein coupled receptor protein signaling pathway	P	5	GO:0007186	18
Cell communication	P	3	GO:0007154	13

Peptidases can degrade host protein material such as collagen, fibrin and actin in the connective tissue of the extracellular matrix, but are also cable of destroying immunoglobulins [42-45]. In this study, we found over 200 transcripts annotated to encode peptidases belonging to five major peptidase clans: serine (45), cysteine (35), metallo (47), aspartate (10), and threonine peptidases (7) (Table 1). Within all peptidases, QPX was found to possess several peptidases commonly associated with pathogenicity. These include papain-type cysteine peptidases (IPR000169; IPR000668, Additional file 1) also known as cathepsins, which are the dominant peptidases in pathogenic protozoans [46]. Further, QPX possesses serine peptidases in the S8 family known as subtilisins or subtilases (IPR000209; IPR015500, Additional file 1), which are universal virulence factors found in pathogenic bacteria [47,48], protozoa [49,50], and fungi. QPX also possesses sequences encoding peptidases found to be virulence factors in other pathogens [44,51-54] including aspartic peptidases (family A1), serine peptidases (families S1, IPR001254; S9, IPR001375; and S10, IPR001563) and metallopeptidases (families M16, IPR011237; M28 and M35) (Additional file 1). Based on the sequence information, QPX is expected to use hydrolytic enzymes, including peptidases, to break down extracellular material of clam tissue to be able to propagate throughout the host body. In situ observation of degraded clam tissues surrounding QPX cells [8,9,48] indirectly supports the involvement of peptidases in the pathogenesis of this parasite.

QPX infection inside the clam tissue leads to an inflammatory response of clam hemocytes aggregating around QPX cells. It is also known that the hemocytes of bivalves, including *M. mercenaria,* produce reactive oxygen species (ROS) to combat invading organisms [55]. To survive the clam defense, QPX would be expected to produce antioxidant molecules which can neutralize the toxic effect of ROS. In the generated QPX transcriptome library, seventeen sequences were identified to be involved in antioxidant activity (GO:0016209, Table 1) including catalases (IPR002226), superoxide dismutases (IPR001424, IPR001189), thioredoxin peroxidases (IPR012336, IPR005746), glutathione peroxidases (IPR000889, IPR002109), and ascorbate peroxidases (IPR010255 IPR002207) (Additional file 1). All of these antioxidant molecules have been shown to be effective against endogenous (metabolism related) and exogenous (from host defense) ROS species and are essential for resistance to oxidative stress in many pathogenic protozoans during disease development [56,57]. QPX antioxidant armor suggests that the pathogen is prepared to overcome *M. mercenaria* hemocyte-derived ROS, and facilitate the establishment of an infection.

During infection, molecules that play the most important roles are the ones on the interface between the host cell or its extracellular matrix and the pathogen cell. These molecules are involved in cell recognition, cell adhesion and cell communication. The QPX library allowed the identification of 34 sequences annotated to molecular function term receptor activity (GO:0004872), 18 linked to G-protein coupled receptor protein signaling pathways (GO:0007186), 13 transcripts involved in cell communication (GO:0007154), and 4 transcripts related to cell adhesion (GO:0007155) (Table 1). These molecules are excellent targets for future research studies on the capabilities of QPX to anchor itself to the extracellular matrix within clam tissue. For example, some adhesive and ligand binding molecules of plant and animal pathogens are related to thrombospondin [58,59], integrin [59,60] and lectins [61-66]. Our results show that the QPX transcriptome contains transcripts with homology to lectins (qpx_c8760, IPR008985; qpx_c13004, qpx_c14444), fibronectin-related proteins (qpx_c14678, IPR003961 and qpx_c7827, IPR008957), integrins (qpx_lrc16604, qpx_lrc4391, qpx_lrc9075), and thrombospodin-related molecules (qpx_c12015) (Additional file 1).

### QPX oligoarray gene expression profiles in response to temperature and QPX adaptation to temperature changes

**Figure 2:**
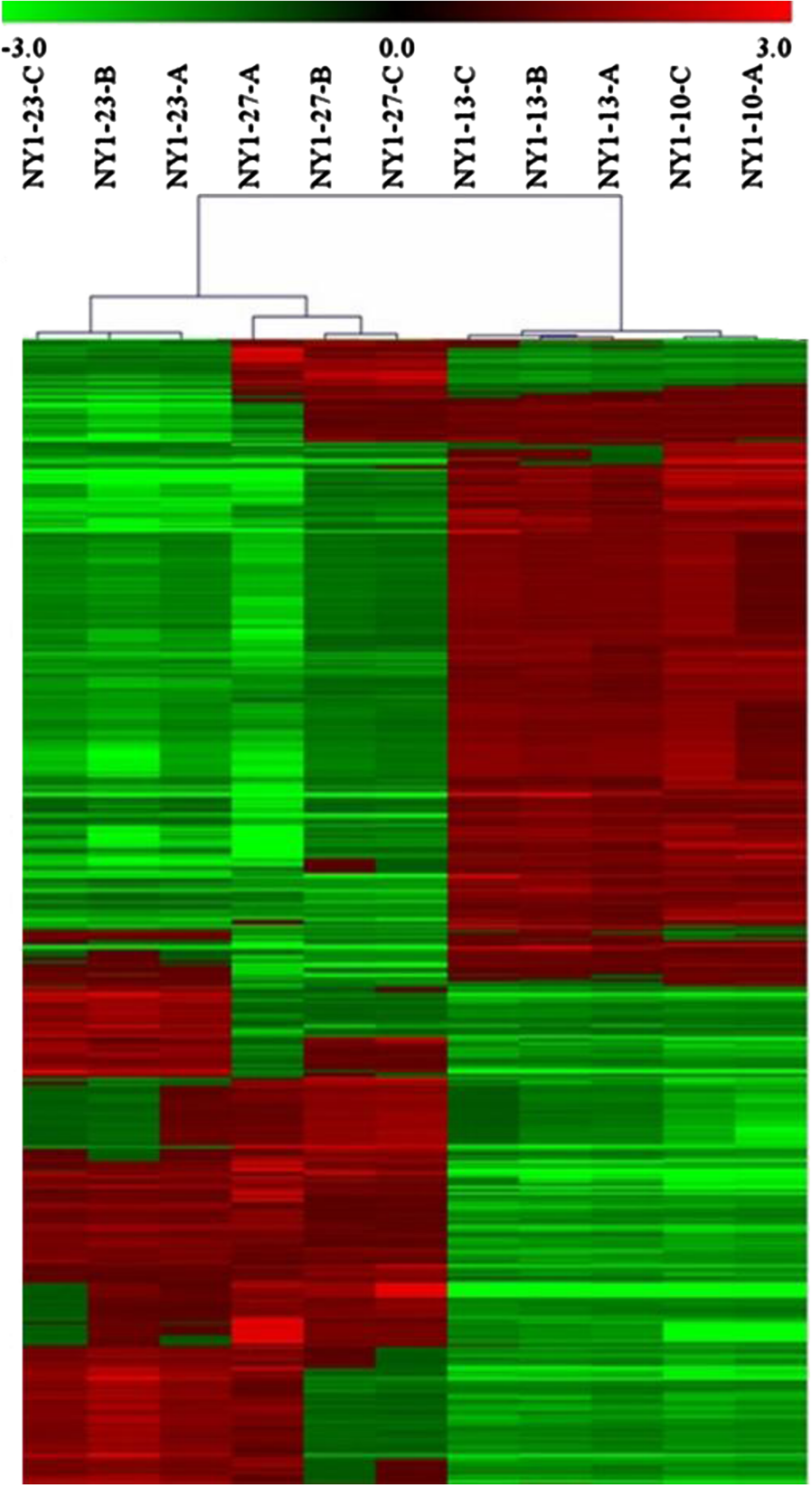
**Heat map of differentially expressed QPX transcripts.** Hierarchical clustering of 1,580 QPX transcripts (Pearson’s correlation centered) identified to be differentially expressed at four temperature treatments by oligoarrays. The clustering of samples within each treatment (27°C, 23°C, 13°C, n = 3 and 10°C, n = 2) highlights the reproducibility of the assay.

**Figure 3:**
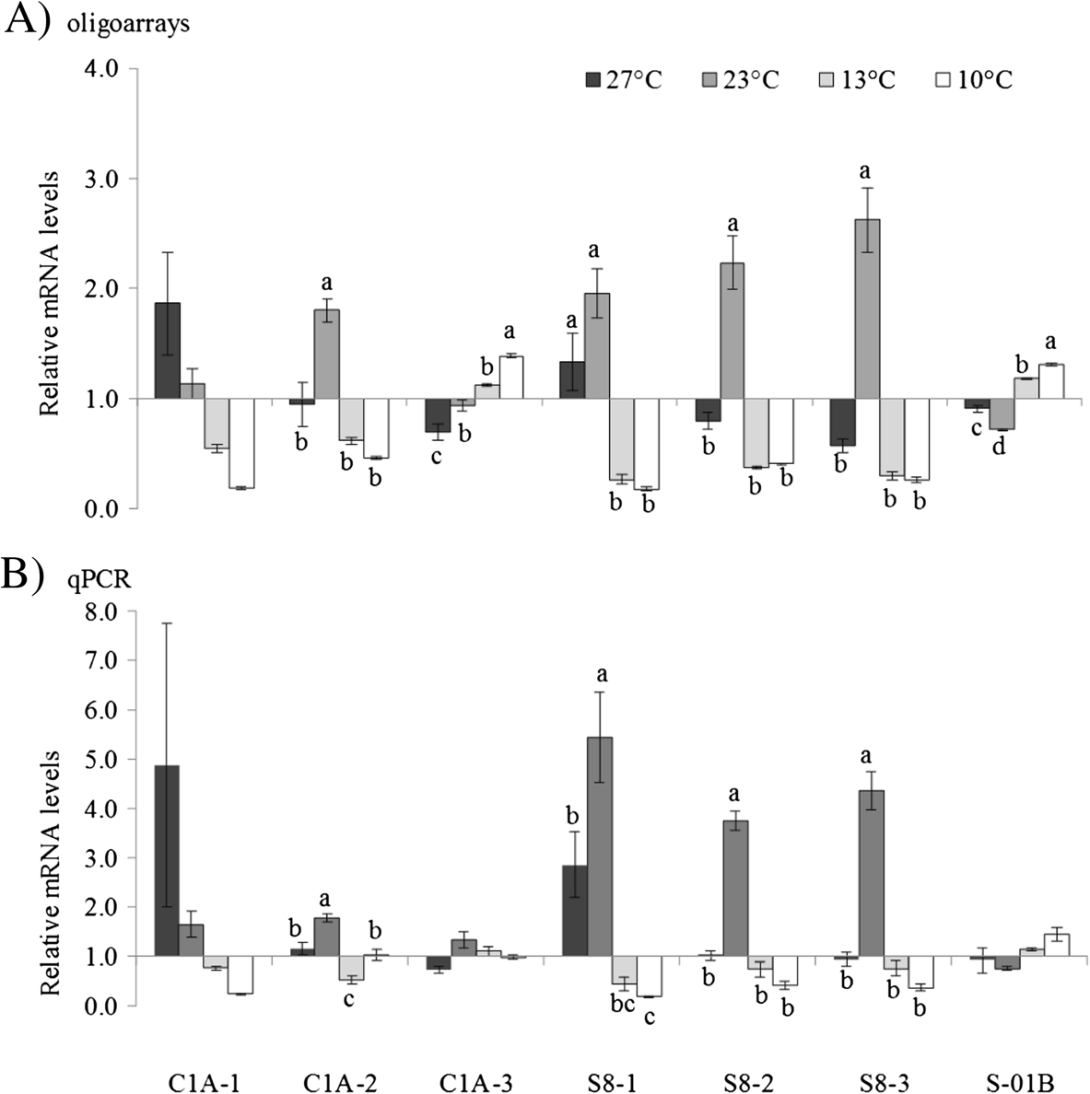
**Validation of oligoarray results.** Relative mRNA expression levels (mean ± std error, n = 3) of six peptidases in QPX grown at four different temperatures as determined by oligoarrays **(A)** and quantitative RT-PCR **(B)**. Significant differences (ANOVA, p < 0.01, Tukey post-hoc test) between treatments for each gene are indicated by letter labels.

The gene expression profiles of QPX cultivated at four different temperatures (27°C, 23°C, 13°C, 10°C) were investigated using 15 K 60-mer oligonucleotide arrays. A total of 1,580 transcripts were differentially expressed (DE, at least 1.5 fold in conjunction to ANOVA p < 0.01) in response to temperature changes (Figure 2, Additional file 2). To confirm gene expression patterns obtained by the oligoarray analysis, transcription levels of seven different QPX peptidases (four serine and three cysteine peptidases: S8-1, qpx_c765; S8-2, qpx_c1822; S8-3, qpx_c2550; S01B, qpx_c674; C1A-1, qpx_c534; C1A-2, qpx_c5487; C1A-3, qpx_c3110; Table 2) at four different temperatures were examined using quantitative real time PCR. Using qPCR method, the mRNA expression patterns of four of the seven examined peptidases altered by the temperature conditions were statistically significant (ANOVA, p < 0.001) and the data was significant for six out of the seven peptidases based on oligoarrays results (Figure 3). The comparison of the relative transcript levels determined by the two methods, oligoarray and qPCR resulted in a strong statistically significant correlation (Pearson, r = 0.86).

**Table 2:** Sequence information for transcripts selected for the quantitative PCR validation of oligoarray data

**Sequence name**	**Sequence ID**	**Sequence description**	**Primer sequences**		**Top blast match**	
					**Acc. no.**	**E-value**
C1A-1	qpx_c534	Cysteine peptidase	F:	ACGGCAATGTTACCGAAGAGGCTA	XP_002507788	1.6E-48
			R:	TAGTTATCCAATGGGCCCGCGTTA		
C1A-2	qpx_c5487	Cysteine peptidase	F:	ACTGGAGCAAGAAGGGAGCAGTAA	CBJ28832	1.9E-29
			R:	AAGACCACCTGTGGTGGAGAAACT		
C1A-3	qpx_c3110	Cysteine peptidase	F:	TGCAGGTCGTCGTTGCTTTAGTCT	EAY93080	3.8E-14
			R:	TAGCCAACGATTGAAACTGCGTGG		
S8-1	qpx_c765	Serine peptidase	F:	TCGTGCTGGACACATAGTTGTCGT	ABI79453	6.3E-30
			R:	TATCGGTGGCTCCAACGCTTATCA		
S8-2	qpx_c1822	Serine peptidase	F:	TATGGCTACTCCATTTGTCGCTGG	ABI79453	1.9E-29
			R:	ACGAGCAAATTGGGAGATTCGTGC		
S8-3	qpx_c2550	Serine peptidase	F:	AAGAGCGGTTGGGAATATGGGAGT	ABI79453	7.5E-51
			R:	AACAACAAGCATGCCCTCTTCTGC		
S01B	qpx_c674	Serine peptidase	F:	AGGTCTACGTATGGCTCCACTTCA	XP_002906640	1.7E-22
			R:	TTGAGCCCACATTTCTCAGCAGGA		
Actin	qpx_c116	Actin	F:	TGAAGATCTTGACCGAGCGTGGTT	ABC85743	1.0E-173
			R:	AGCGGTCTTCATCTCCTGGTCAAA		

**Figure 4:**
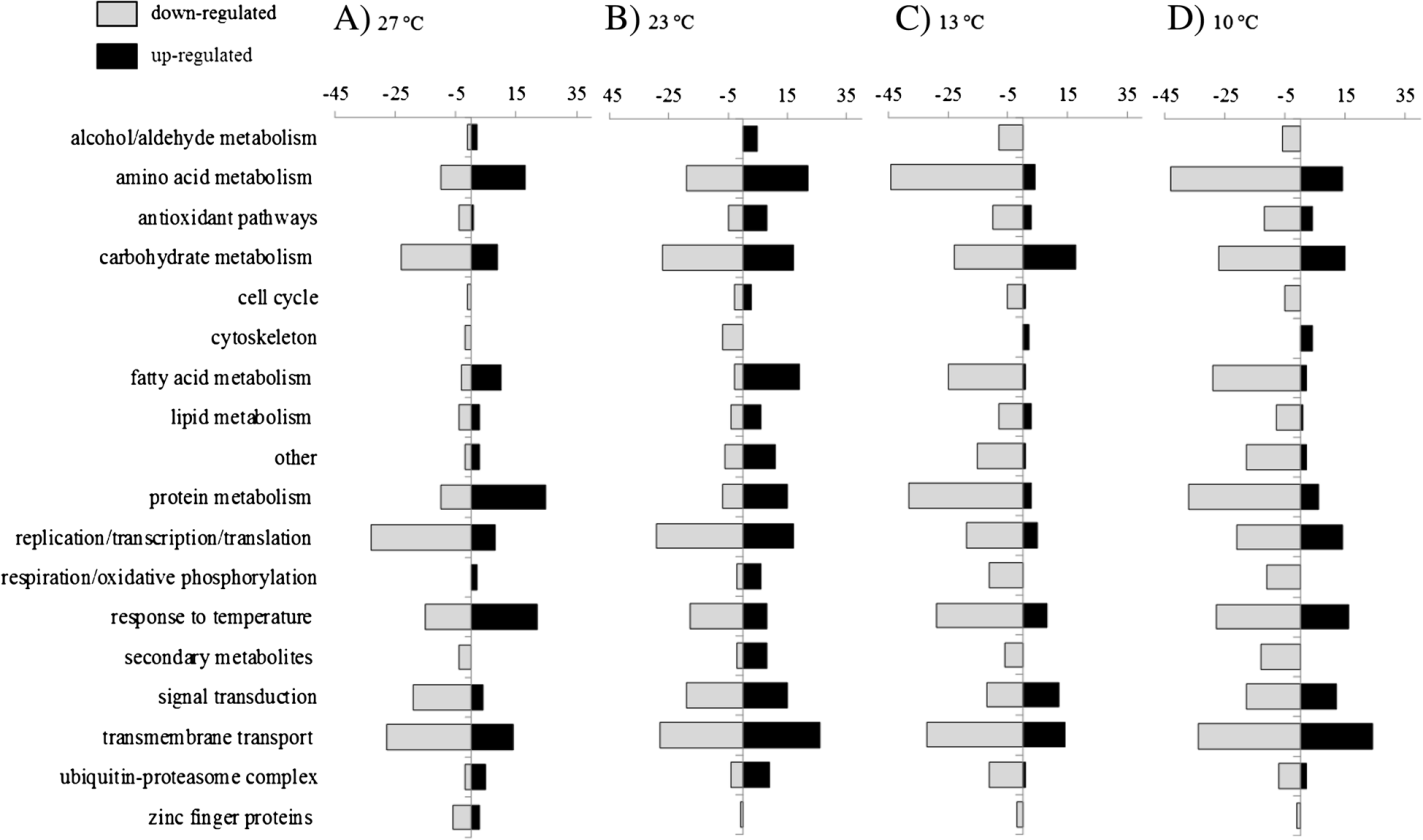
**Molecular processes affected by temperature treatments.** Number of annotated QPX transcripts differentially expressed (DE) in response to four temperature treatments (A: 27 °C, B: 23 °C, C: 13 °C, D: 10 °C). Transcripts are grouped by their putative biological process and molecular function into 17 operational groups.

Among the 1,580 DE transcripts, only 617 had known predicted biological or molecular function (Additional file 2) and a subset of these is presented in Table 3. The GO annotations, as well as the information on the best blast hit result for the remaining sequences are given in Additional file 2. These 617 sequences were grouped into 17 functional categories (Figure 4) to identify biological processes differentially expressed under different temperature treatments. In general, the highest number of differentially expressed genes included those involved in cell metabolic processes such as amino acid, carbohydrate, protein, fatty acid and lipid metabolism (241 transcripts in total, Figure 4). This pattern, however is biased by the fact that public databases are skewed toward most conserved genes which tend to be genes belonging to the metabolic pathways. More than half of the QPX genes regulated in response to temperature have unknown molecular functions (61%, Additional file 2).

**Table 3:** Selected differentially expressed genes in QPX exposed to four temperature treatments

**Sequence description**	**Mean fold changed**				**Seq. ID number**	**Top BLAST Hit**	
	**27°C**	**23°C**	**13°C**	**10°C**		**Accession no.**	**E-value**
**Protein stability**							
dnaj protein 1	-1.5	-1.6	1.4	1.2	qpx_c101	XP_002287676	4.13E-06
dnaj protein 2	-1.3	-2.3	1.4	1.5	qpx_c9231	XP_002505603	7.23E-23
dnaj protein 3	-2.1	-1.9	1.5	1.6	qpx_c11973	EGZ29544	1.71E-06
Heat shock protein 40 kDa	-2.2	-1.7	1.4	1.6	qpx_c37	XP_003496481	1.35E-41
Heat shock protein 70 kDa 1	-2.7	-4.1	1.6	1.9	qpx_c366	XP_002788182	1.24E-33
Heat shock protein 70 kDa 2	-2.2	-3.1	1.6	1.6	qpx_c2869	EGZ24428	4.92E-79
Heat shock protein 70 kDa 3	-1.7	-1.8	1.4	1.4	qpx_c1020	XP_001694468	9.67E-58
Heat shock protein 90 kDa	-0.9	-4.0	1.5	1.6	qpx_c16851	XP_002291118	6.50E-49
Heat shock protein 100 kDa	0.2	-3.8	1.7	0.1	qpx_c3466	XP_635137	5.11E-27
Heat shock protein 18 kDa	2.2	0.5	-5.5	-6.0	qpx_c56	NP_662846	1.95E-16
Heat shock protein 16 kDa	0.3	1.8	-1.9	-2.2	qpx_c2700	ADR66511	4.49E-15
Heat shock protein 15 kDa	0.4	1.5	-1.6	-1.6	qpx_c5637	YP_969657	1.64E-13
**Proteolysis**							
Cysteine peptidase C1-1	1.9	0.5	-1.9	-5.3	qpx_c534	XP_002507788	1.07E-56
Cysteine peptidase C1-2	0.2	1.8	-1.6	-2.2	qpx_c5487	XP_002178071	6.01E-34
Cysteine peptidase C1-3	-2.1	-1.3	0.4	2.0	qpx_c4154	CAB43538	1.44E-09
Cysteine peptidase C1-4	-3.9	4.6	-5.5	-2.7	qpx_c1221	EGD76061	1.37E-06
Cysteine peptidase C1-5	-2.2	-2.3	1.5	1.6	qpx_c8710	XP_003212177	2.04E-13
Metallopeptidase M12B	-5.9	-5.9	1.8	2.3	qpx_c6353	ADW54356	3.64E-06
Metallopeptidase M32	-1.7	-1.4	1.6	1.1	qpx_c5681	ZP_09027479	5.64E-21
Serine peptidase S8-1	0.7	2.0	-3.9	-5.7	qpx_c765	ABI79453	1.82E-34
Serine peptidase S8-2	-1.3	2.2	-2.7	-2.5	qpx_c1822	ABI79453	3.26E-34
Serine peptidase S8-3	-1.8	2.6	-3.5	-3.8	qpx_c2550	ABI79453	2.46E-53
Serine carboxypeptidase S10-1	0.3	1.7	-2.0	-1.7	qpx_c2447	EGB09909	1.21E-47
Serine carboxypeptidase S10-2	0.7	2.0	-2.8	-2.7	qpx_c8079	XP_001747631	8.39E-08
Serine carboxypeptidase S28-1	0.3	1.5	-1.4	-1.4	qpx_c13341	CCA21035	1.27E-13
Serine carboxypeptidase S28-2	-0.2	1.8	-2.2	-2.8	qpx_c2535	EGD81876	6.83E-53
**Oxidative stress**							
Catalase	-1.6	-2.1	1.2	1.9	qpx_c2181	AEX91749	1.50E-33
Cu/Zn superoxide dismutase 1	-0.6	1.9	-1.7	-2.0	qpx_c1801	AAN85727	6.97E-28
Cu/Zn superoxide dismutase 2	-0.3	1.7	-1.7	-1.8	qpx_c3152	ADN04915	9.10E-40
Mg/Fe superoxide dismutase	-0.4	1.9	-2.2	-1.9	qpx_c6686	XP_002958078	2.13E-27
Superoxide NADPH oxidase	-1.8	-1.8	1.5	1.5	qpx_c1054	EGB04413	5.01E-24
Thioredoxin 1	-0.4	1.9	-1.9	-2.2	qpx_c1718	XP_765168	4.11E-25
Thioredoxin 2	-1.2	1.6	-1.4	-1.3	qpx_c2033	XP_002402396	3.08E-27
Thioredoxin 3	-0.2	1.9	-2.2	-2.5	qpx_c4189	XP_001800478	8.60E-19
Thioredoxin reductase 1	0.4	-1.2	-1.2	1.5	qpx_c1123	EEE25525	2.45E-38
Thioredoxin reductase 2	-1.8	-2.0	1.5	1.4	qpx_c2283	EGB06390	7.90E-46
Thioredoxin reductase 3	1.1	1.3	-1.2	-1.6	qpx_c3882	XP_002181543	4.13E-33
**Polysaccharide synthesis and secretion**							
Glycosyltransferase	-0.8	-2.4	1.4	1.4	qpx_c2871	XP_002296954	3.04E-05
Bligosaccharyl transferase	-1.4	-2.2	1.4	1.5	qpx_c5264	XP_002185367	2.06E-22
Beta-amylase/glucosidase	-4.2	-1.2	1.3	1.8	qpx_c1194	ZP_08507128	2.84E-13
Alpha-amylase/glucosidase	-4.9	-4.8	1.7	2.2	qpx_c114	YP_750476	1.26E-32
Sugar transporter 1	-1.5	-1.4	1.3	1.5	qpx_c6741	XP_003452761	1.54E-13
Sugar transporter 2	-3.4	-2.1	1.5	2.0	qpx_c730	XP_642887	2.37E-12
gdp-mannose-3-epimerase	-1.5	-1.1	1.2	1.3	qpx_c4458	XP_002956437	8.07E-94
udp-galactose-4-epimerase	0.1	-3.1	1.4	1.5	qpx_c5964	ZP_08151628	5.96E-34
udp-glucose gdp-mannose dehydrogenase	-7.9	-5.1	2.5	1.2	qpx_c15700	ZP_06587676	1.90E-28
udp-glucose 6-dehydrogenase	-2.6	-3.7	1.7	1.9	qpx_c7532	CBJ28343	1.02E-25
Integrin-related protein 1	-2.0	-1.4	1.5	1.5	qpx_c5688	EGB09976	3.30E-07
Integrin-related protein 2	-2.2	-2.2	1.6	1.5	qpx_lrc9075	ZP_08823332	9.08E-08
Syntaxin	-2.5	-2.6	1.6	1.8	qpx_c324	EGB05290	1.40E-15

Mean fold changes are shown (3 biological replicates).

The patterns of DE metabolic process vary between the temperature treatments with the most noticeable difference showing suppression of metabolic processes at cold temperatures: 178 transcripts down regulated at 13°C and 184 down regulated at 10°C in comparison to 79 transcripts down regulated at 27°C and 88 at 23°C (Figure 4). The slowing down of metabolism is a common response of ectothermic organisms to cold temperatures [67]. In addition, results suggest that the cold temperature conditions lowered the respiration rate of QPX (Figure 4) supporting a lower metabolic rate for the parasite. In contrast, seven transcripts within the respiration and oxidative phosphorylation process were induced at 23°C, suggesting an increased respiration rate at this temperature. The enrichment analysis revealed just a few biological and molecular processes being affected by the temperature changes but not in all temperature treatments (Table 4). For instance, the histidine catabolic processes were enriched by 151 fold among genes up-regulated at 27°C, while cellular aldehyde metabolic processes were enriched by 49 fold among genes up-regulated at 23°C and by 69 fold among genes down-regulated at 10°C (Table 4). These changes reflect the adjustment of metabolic rate of the parasite at different temperatures as shown by the overall patterns presented in Figure 4.

**Table 4:** Gene Ontology terms enriched in response to temperature changes (Fisher’s exact test with multiple testing correction of FDR < 0.05, Benjamini and Hochberg)

**GO term ID**	**Gene ontology term description**	**GO type**	**FDR**	**p-value**	**% in DE group**^**a**^	**% on array**^**b**^	**Fold enrich**^**c**^
**Up-regulated at 27°C**							
GO:0006548	Histidine catabolic process	P	2.9E-02	2.8E-05	5.4	0.0	151
GO:0016829	Lyase activity	F	2.5E-02	8.0E-06	19.6	3.7	5
**Down-regulated at 27°C**							
GO:0019915	Lipid storage	P	1.8E-02	2.2E-05	3.7	0.0	104
GO:0005840	Ribosome	C	2.9E-02	5.7E-05	11.1	2.0	6
GO:0003735	Structural constituent of ribosome	F	1.8E-02	2.9E-05	11.1	1.8	6
**Up-regulated at 23°C**							
GO:0006081	Cellular aldehyde metabolic process	P	2.5E-02	3.2E-05	3.5	0.1	49
GO:0003755	Peptidyl-prolyl cis-trans isomerase activity	F	1.3E-02	9.7E-06	4.4	0.1	30
GO:0000502	proteasome complex	C	1.3E-02	1.2E-05	5.3	0.3	16
**Down-regulated at 10°C**							
GO:0006548	Histidine catabolic process	P	8.1E-03	1.0E-05	2.4	0.0	69
GO:0008233	Peptidase activity	F	4.9E-02	1.7E-04	11.0	3.9	3
GO:0016829	Lyase activity	F	4.9E-02	1.9E-04	10.4	3.6	3
GO:0006081	Cellular aldehyde metabolic process	P	4.9E-02	1.4E-04	2.4	0.1	33
GO:0016616	Oxidoreductase activity, acting on the CH-OH group of donors, NAD or NADP as acceptor	F	2.7E-02	4.3E-05	6.1	1.1	6

^a^Percent of sequences with that gene ontology term in the differentially expressed gene group.

^b^Percent of sequences with that gene ontology term on the whole array.

^c^Fold enrichment: Fold increase of the GO term in the differentially expressed gene group.

The 23°C is in a range of QPX optimal growth conditions, however this temperature condition was also associated with an up-regulation of genes related to oxidative stress (eight up regulated transcripts, Figure 4). Slight oxidative stress, characterized as an accumulation of reactive oxygen species (ROS), happens in every cell as a result of imbalance between oxygen availability and oxygen consumption from respiration [67]. If a small amount of ROS, specifically superoxide anion, is produced during the normal respiration process as a simple by-product, an increased respiration rate would cause increased production of ROS. QPX possesses six different sequences encoding superoxide dismutase enzymes (SODs, data not shown), including four copper/zinc related SODs which are usually found in the cytosol of a cell, and two iron/manganese SODs which are usually found in mitochondria. The expression of only three QPX SODs (two Cu/Zn SOD and one Fe/Mn enzymes) were up-regulated at 23°C, suggesting that these three SODs are the main enzymes expressed in QPX cells to cope with the accumulation of endogenous ROS during the normal respiration process.

A well-documented response of organisms to temperature change is the regulation of expression of molecular chaperones known as heat shock proteins which are responsible for maintaining protein stability in situ. Temperature stress can lead to changes in proteins configuration and their potential malfunction but heat shock proteins control aggregation of denatured proteins in the cell, and are responsible for their reconfiguration, translocation across membranes and degradation. Overall 49 QPX transcripts coding for heat shock proteins were found to be regulated with temperature changes (Figure 4, Additional file 2). The amino acid sequence alignments of theses transcripts show that QPX has three different heat shock proteins which can be classified into a group of molecular chaperones of low monomeric molecular mass, ranging from 12 to 43 kDa [68]. These low molecular weight chaperones were up-regulated at the elevated temperature of 27°C (heat shock proteins 15, 16 and 18 kDa, Table 3). The transcription levels of these small molecular weight chaperones have been commonly investigated in pathogenic protozoans affecting humans and their expression is usually regulated during heat stress related to fever (e.g. *Trypanosoma cruzi*[68], *Toxoplasma gondii*[69]). Nine molecular weight chaperons: three dnaj-like proteins, three heat-shock protein 70, one heat-shock proteins 40, one 90 and one 101 kDa (Table 3) were up-regulated in response to cold temperature conditions revealing that they fulfill a role of cold shock chaperons. Heat shock proteins 70 and 90 have been previously documented to act as cold temperature chaperons in bacteria [70], nematode larvae [71], and oomycetes [72]. The dnaj proteins are co-chaperones of hsp70 and contain a conserved amino acid J domain which binds to hsp70 and stabilizes the interaction between the substrate and hsp70 [73]. Their up-regulation in QPX at low temperature highlights a similar response to cold stress and underlines the ability of the parasite to cope with suboptimal temperatures.

### QPX putative virulence factors regulated by temperature

Temperature is one of the environmental factors controlling QPX disease development in hard clams [11,12] and one of the goals in the present study was to investigate the response of QPX putative virulence factor to temperature changes. Overall 14 QPX peptidases were found to be regulated with temperature changes including seven serine peptidases, five cysteine peptidases, and two metallopeptidases (Table 3). One papain-like peptidase (qpx_c534) was up-regulated at 27°C, two (qpx_c5487, qpx_c1221) were up-regulated at 23°C, and two (qpx_c4154, qpx_c8710) were up-regulated at 13°C and 10°C (Table 3), suggesting different roles for these genes in basic metabolism and/or pathogenesis of the parasite. Papain-like cysteine peptidases are some of the most extensively studied virulence factors. They play a crucial role in parasite biology, including growth, development and replication. They are also implicated in host tissue degradation, including digestion of host extracellular matrix proteins and destruction of host immune-related proteins such as immunoglobulins [74]. Further specific experimental studies are needed to specifically characterize the biochemical function and role of each papain-like peptidase in QPX biology and virulence abilities. Other QPX peptidases for which expression was shown to be regulated at different temperatures were subtilases. The amino acid sequence alignments of QPX transcripts annotated as belonging to the S8 family revealed that QPX possesses at least 6 different subtilisin-like peptidases (data not shown). Only three subtilases (qpx_c765, qpx_c1822, qpx_c2550) were shown to be differentially expressed in response to temperature changes, with the highest levels measured at 23°C (Figure 3, Table 3) which corresponds to QPX’s optimal growth temperature. QPX is a saprophytic microorganism which needs to degrade nutrient proteins extra-cellularly and transport oligopeptides and amino acids into the cell via diffusion. QPX maximal growth at 23°C could then explain and justify the need for an efficient extracellular protein degradation and nutrient acquisition system associated with high expression of its digestive enzymes. Subtilases, however are also universally occurring secreted enzymes found in many medically important pathogenic microorganisms, including bacteria (e.g. *Mycobacterium*, *Streptococcus*[47,48,75-77]), protozoa (e.g. *Plasmodium*, *Leshmania* and *Toxoplasma*[50,78-80]), and fungi (e.g. *Aspergillus* spp. [81]). Therefore, the role of subtilisins as QPX virulence factors might also be a possibility, but these enzymes may be important for host tissue digestion and disease progression during advanced and late stages of infection when the overcoming of host immune system is less important but degradation of the tissue and pathogen proliferation takes over.

The M12B peptidase (qpx_c6353, Table 3) which was up-regulated at 13°C and 10°C also require special attention. It belongs to the ADAMs or A Dis-integrin And Metalloprotease family of zinc-dependent endopeptidases. The same QPX peptidase was also found to be regulated with temperature in another study [28]. In animals, ADAMs are membrane-anchored glycosylated enzymes capable of degrading proteins from the surface of cells, thus playing important roles in cell adhesion, signaling, cell-cell fusion, and cell-cell interactions [82,83]. They possess two different protein components in addition to the metalloprotease domain, including an integrin domain (with adhesion and receptor activities) and a cytosolic domain which provides an attachment for various signal transduction proteins [82]. Cell-surface glycol-metallo-proteases play an important role in the pathogenesis of trypanosomatids, as they allow these parasites to adhere and move through connective tissue of their hosts [84]. The specific role, if any, of adamysin-like protease in the virulence of the QPX requires additional studies.

The QPX M32 enzyme is a carboxy-Taq-metallopeptidase (qpx_c5681, Table 3). The first zinc-containing thermostable metallopeptidase was originally discovered and purified from *Thermus aquaticus* and had an optimal enzymatic activity at 80°C [85]. Most bacterial and archaebacterial species are known to possess the M32 carobxypeptidases, but they are considered absent in most eukaryotic genomes except for a few protozoan species [86]. Their biological function in these organisms remains unknown. However, the basic characteristics of two recombinant M32 peptidases of *Trypanosoma cruzi* have been investigated [86]. They were shown not to behave as themostable enzymes, with their activity significantly decreasing with increased temperature. The current study is the first report of M32 enzyme in a thraustochytrid species for which stability and activity increases at lower temperatures. This cold resistant QPX’s metallopeptidase is a very interesting target for investigation of its biological function and role in the infection process.

QPX cells release a gel-like muco-filamentous secretion which appears to protect QPX from clam hemocyte phagocytosis and encapsulation as seen during histo-pathologic observation of infected clam tissues [2,8,9,87]. In addition, QPX mucus causes necrosis of clam hemocytes in vitro [88] and provides protection against clam humoral defense [89]. Overall, the generated transcriptome library contains 21 sequences involved in polysaccharide metabolic processes (GO:0005976) including 13 sequences involved in the biosynthesis of polysaccharides (GO:0000271) but complete pathways of mucus production were not identified (Table 1). The consistency of QPX mucus secretion changes with different temperatures, from a gel with low viscosity at 27°C to a very rigid gel at 10°C, thus it is surmised that cold temperatures would benefit the parasite by providing stronger protection during the infection process. Thicker mucus secretion at lower temperature was associated with an up-regulation of GDP-mannose dehydrogenase (qpx_c15700, Table 3). This enzyme essentially catalyzes irreversible conversion of GDP-mannose to GDP-mannuronic acid and ultimately leads to the biosynthesis of alginates in *Pseudomonas aeruginosa*[90] and in the brown alga *Ectocarpus siliculosus*[91]. In addition, the expression of two enzymes involved in the production of mannose (gdp-mannose-3- epimerase) and galactose (udp-galactose-4-epimerase) were also up-regulated at the two cold water temperatures (qpx_c4458 and qpx_c5964, respectively, Table 3). Mannose is the most important monosaccharide required for the production of mannuronic acid, which is the major component of alginates [92]. Interestingly, the mRNA levels of several different transcripts coding for UDP-glucose/GDP-mannose dehydrogenase were up-regulated in QPX at the two cold temperatures (qpx_c7532 in Table 3, full list in Additional file 2). Further, there were also a few other molecules in the carbohydrate metabolism and vesicular secretion pathways that were induced at 10 and 13°C. These include two glycosyltransferases (qpx_c2871, qpx_c5264, Table 3) which transfer and add a range of different sugars to other sugars, phosphates and proteins, thus participating in glycosylation of proteins and in synthesis of polysaccharides and glycoconjugates [93]. If the up-regulated glycotransferases are involved in the glycosylation process of QPX extracellular mucopolysaccharides, it can provide additional support of the hypothesis of considerable changes to the QPX mucus structure at cold temperature. In addition, two sugar transporters (qpx_c730 and qpx_c6741, Table 3) which carry dissolved simple sugars across membranes in response to chemosmotic gradient [94], and a syntaxin-type protein (qpx_c324, Table 3) that is a membrane protein participating in the vesicular membrane fusion during exocytosis [95], were over-expressed at the colder temperatures. Again, it can be speculated that these molecules participate in mucus polymers production and/or rearrangements. Even though the composition and structure of QPX mucus is still to be determined, these findings identify specific candidates that may be involved in mucus biosynthesis and provide a plausible scenario for the regulation of mucus consistency at different temperatures. The present hypothesis that QPX mucus production is increased in cold conditions can also be supported by the up-regulation of several molecules in carbohydrate synthesis and secretion pathways.

## Conclusions

In conclusion, this study characterized the transcriptome of a parasitic labyrinthulid, offering new insights into the molecular bases for the pathogenicity of these microorganisms. The gene ontology annotation completed in this study is an excellent source of information for future investigations of this parasite. Results from the oligoarray experiment demonstrated the ability of QPX to cope with a wide range of environmental temperatures, including those considered to be suboptimal for clam immunity (low temperature), providing a mechanistic scenario for disease distribution in the field and for high disease prevalence and intensity at low temperature. These results will serve as a basis for studies aimed at thorough characterization of specific putative virulence factors.

## Methods

### Library construction and sequencing

QPX isolate NY0313808BC7 [96] initially isolated from a diseased clam collected from Raritan Bay, New York, was used for RNA extraction and library construction. To enhance library coverage, QPX was separately grown in Minimum Essential Medium (MEM, Sigma) supplemented with one of the following protein sources: clam muscle homogenates (500, 1000 or 3000 ug ml-1 [97], fetal bovine serum (0%, 2.5%, 5% or 10%) or gelatin (0.1%, 0.2 or 0.3%). Additional cultures were grown in MEM supplemented with yeastolate (0.1%, 0.2% or 0.3%) or with clam muscle homogenates made in sterile seawater without MEM. Cultures were incubated in triplicate at 23°C and parasite cells were separately harvested on Day 8 and Day 14. Parasite biovolume in each sample was assessed using a fluorometric technique [98] before samples were pooled using the same parasite biovolume from each culture condition. Total RNA was extracted from pooled samples using Trizol (Molecular Research Center, Inc.). The quality of total RNA was verified using Agilent 2100 bioanalyser and quantified by assessing the A260/280 and A260/230 ratios using a Nanodrop (ND 1000) spectrophotometer. Poly(A+) RNA were isolated from 300 μg of total RNA using the PolyATract® mRNA Isolation system (Promega) following the manufacturer’s instructions and used for cDNA synthesis. Preparation and sequencing of the cDNA library were performed at the McGill University and Génome Québec Innovation Centre (Canada) following the manufacturer’s protocol (Roche-454 Life Sciences, Brandford, CT, USA). The SMART adaptor sequences were removed from reads using a Perl script. Reads less than 50 bp and low-quality (quality scores below 20) reads were filtered out using SeqClean and remaining high-quality reads were assembled using MIRA assembler (version 3.0.5) using the default parameters for de novo EST assembly of 454 reads [99].

### Transcriptome annotation

The annotation of the QPX transcriptome was completed using the online bioinformatics tool Blast2GO [100]. First, all sequences were subjected to blastx (basic local alignment tool) searches against the National Center for Bioinformatics (NCBI) sequence database with E-value cut off of 10^-3. Next the sequences proceeded through mapping to Gene Ontology (GO) Consortium database of standardized phrases describing functional information of known gene products, and finally GO functional annotation was completed using cutoff value 10^-3. Using the same tool, the sequences in all six translation frames were subjected to the InterProScan to find conserved protein domain matches in the Integrated Protein database of the European Bioinformatics Institute [101].

### *In silico* identification of QPX putative virulence factors

The selection of potential virulence factors was based on the above annotated QPX transcriptome library and homology of QPX transcripts and protein sequences to the virulence factors of other protistan parasites and pathogens. The QPX sequence library was screened for sequences encoding for a variety of peptidases, hydrolytic enzymes, antioxidants, cell surface receptor and adhesion molecules. Manual curation of automated annotation as well as alignments and translations into amino acid sequences was accomplished using Geneious software [102]. The translated amino acid sequences were manually checked for the correct protein signatures and conserved protein domain using MEROPS, the peptidase database at http://merops.sanger.ac.uk/[103] and in the InterPro database from the European Bioinformatics Institute, http://www.ebi.ac.uk/Tools/pfa/iprscan5/[101,104].

### Transcriptomic changes in QPX in response to temperature

For the temperature treatment, 500 μl of an exponentially growing QPX (isolate NY0313808BC7) culture was inoculated into twelve 25-ml culture flasks (Falcon) filled with 5 ml of Minimum Essential Medium (MEM, Sigma) supplemented with 10% of fetal bovine serum (FBS, Sigma). The flasks were incubated at four different temperatures: 27°C, 23°C, 13°C and 10°C, in triplicates (except 10°C which was done in duplicate) for each treatment. After seven days of incubation, the cultures were diluted with equal volume of filter sterilized artificial seawater and passed several times through a syringe to facilitate liquefaction of QPX mucus secretion. The mixtures were then transferred into 15-ml conical tubes and centrifuged at 3000 g for 40 minutes at 4°C. The supernatant was discarded and cell pellets collected and kept on ice for immediate RNA extraction. Trizol reagent (Molecular Research Center, Inc.) was used to isolate RNA from all samples following manufacturer’s protocol. RNA quality and quantity were estimated spectophotometrically using a Nandrop spectrophotometer.

### Oligoarray design and hybridization

A subset of contigs produced by MIRA was used for the production of a 8 × 15 k 60-mer oligonucleotide array using the Agilent eArray application (https://earray.chem.agilent.com/earray/). These included 6,781 curated annotated sequences and 8,297 non-annotated sequences (minimal size = 215 b) to emphasize gene discovery. One probe was produced for each submitted sequence. Probes were synthesized in situ along with positive and negative controls using 8x15K-feature Agilent format slides. Labeled (Cy3 or Cy5) complementary RNA (cRNA) was synthesized from 150 ng of RNA purified from cultures submitted to different temperatures using Trizol (Invitrogen) and the Two-Color Microarray-Based Gene Expression Analysis Protocol (Quick Amp Labeling) following manufacturer’s protocol. Labeled cRNA was purified using Illustra CyScribe GFX Purification Kit (GE Healthcare). cRNA quantity and quality (including dye incorporation) were determined by spectrophotometry (Nanodrop). Samples were considered satisfactory if cRNA concentration and incorporation efficiency exceeded 300 ng/µl and 8 pmol Cy/µg cRNA, respectively. All arrays were hybridized with the same amount of cRNA (300 ng of each Cy3- and Cy5-labeled cRNA). Arrays hybridization and washes were conducted according to the kit protocol and the arrays were scanned with a GenePix 4000B scanner (Molecular Devices, Sunnyvale, CA, USA) using the suggested Agilent scan settings.

### Oligoarray data normalization and analysis

Spot fluorescence intensities were extracted using GenePix software. LIMMA package in R software was used to normalize the intensities data and to remove within-array (method: global lowess) and between-array (method: quantile) non-biological variation [105]. After normalization, the intensities in separate color channels were exported into an excel spreadsheet for further data quality control and trimming. The intensities which were less than two-fold background intensities were eliminated from further analysis. The relative expression of each transcript at each temperature treatment was calculated as the ratio of the mean intensity (n = 3 for 27°C, 23°C, 13°C, and n = 2 for 10°C) for each temperature treatment and the mean intensity of that transcript in all temperature treatments (n = 11). Hierarchical clustering of all samples and genes (Pearson correlation) and the determination of statistically significant differentially expressed genes (one way ANOVA, p-value < 0.01) were completed in the TM4-suite using MeV program [106,107]. The final criteria for differential gene expression were the significance by ANOVA analysis and a one and half fold increase from the mean (up-regulation) or a one and half fold decrease from the mean (down-regulation) in at least one of the experimental treatments. The transcript sequences in the few categories of interest were investigated further by amino acid sequence alignment analysis to identify the number of unique QPX genes responsive to temperature. All translations and alignments were accomplished using Geneious software.

### Real time PCR and oligo array validation

Real time PCR was performed on selected transcripts of interest which were shown to be differentially and no-differentially expressed by oligoarray methodology. Total RNA (2.5 µg) from each sample was used to synthesize cDNA using the MMLV reverse transcription kit (Promega) and oligo dT primers following manufactures protocol. Relative quantification was carried out in 10-µl reactions with Brilliant II SYBR green qPCR master mix (Agilent), 100 nM final primer concentration and 5 ng of RNA-equivalent cDNA. The PCR reactions were performed using Mastercycler ep realplex PCR machine (Eppendorf). Primers with a melting temperature of 60°C were designed using PrimerQuest program (Integrated DNA Technologies, IDT) within the open reading frames encoding for six different QPX peptidases and actin which was used as a reference gene for mRNA levels normalization (Table 2). The amplification products (135 – 148 bp) were confirmed using gel electrophoresis. The peptidases expression levels were normalized to the actin gene and relative transcript levels were calculated using the delta delta Ct method [108].

### Availability of supporting data

This Transcriptome Shotgun Assembly project has been deposited at DDBJ/EMBL/GenBank under the accession GALJ00000000.

http://www.ncbi.nlm.nih.gov/nuccore/GALJ00000000

## Competing interests

The authors declare that they have no competing interests.

## Authors’ contributions

BA, ER and AT designed the study. ER, MP, AT, EPE and BA carried out the experiments. ER and BA analyzed the data and drafted the manuscript. All authors read and approved the final manuscript.

## Supplementary Material

Additional file 1 **Annotation of QPX transcriptome.** This is a Microsoft Excel worksheet that contains information on 17934 transcripts of quahog parasite unknown (QPX). The table contains the top blastx match accession number and E value, Gene Ontology terms and InterPro domain numbers associated with each QPX sequence. Click here for file

Additional file 2 **A list of 1580 QPX transcripts differentially expressed (DE) in response to four temperature treatments.** The file is a Microsoft Excel worksheet that contains a list of 1580 DE QPX transcripts and the mean fold change value for each transcript at each temperature treatment. Click here for file
